# Short clinical crowns (SCC) – treatment considerations and techniques

**DOI:** 10.4317/jced.50556

**Published:** 2012-10-01

**Authors:** Ashu Sharma, G. R. Rahul, Soorya T. Poduval, Karunakar Shetty

**Affiliations:** 1 BDS, MDS. Department of Prosthodontics, Bangalore Institute of Dental Sciences and Research Center, 5/3 Hosur Main Road, Opposite Lakkasandra Bus Stop. Wilson Garden, Bangalore, India.; 2 BDS, MDS. Professor and Head of Department of Prosthodontics. Bangalore Institute of Dental Sciences and Research Center, 5/3 Hosur Main Road, Opposite Lakkasandra Bus Stop. Wilson Garden, Bangalore, India.; 3 BDS, MDS. Professor. Department of Prosthodontics. Bangalore Institute of Dental Sciences and Research Center, 5/3 Hosur Main Road, Opposite Lakkasandra Bus Stop. Wilson Garden, Bangalore, India.

## Abstract

When the clinical crowns of teeth are dimensionally inadequate, esthetically and biologically acceptable restoration of these dental units is difficult. Often an acceptable restoration cannot be accomplished without first surgically increasing the length of the existing clinical crowns; therefore, successful management requires an understanding of both the dental and periodontal parameters of treatment. The complications presented by teeth with short clinical crowns demand a comprehensive treatment plan and proper sequencing of therapy to ensure a satisfactory result. Visualization of the desired result is a prerequisite of successful therapy. This review examines the periodontal and restorative factors related to restoring teeth with short clinical crowns. Modes of therapy are usually combined to meet the biologic, restorative, and esthetic requirements imposed by short clinical crowns. In this study various methods for treating short clinical crowns are reviewed, the role that restoration margin location play in the maintenance of periodontal and dental symbiosis and the effects of violation of the supracrestal gingivae by improper full-coverage restorations has also been discussed.

** Key words:**Short clinical crown, surgical crown lengthening, forced eruption, diagnostic wax up, alveoloplasty, gingivectomy.

## Introduction

A short clinical crown is defined as any tooth with less than 2 mm of sound, opposing parallel walls remaining after occlusal and axial reduction (1). A comprehensive treatment plan and proper sequencing of therapy is needed to overcome the complications presented by a short clinical crown. This paper examines the periodontal and restorative factors related to restoring teeth with short clinical crowns and also various methods available for treating short clinical crowns are reviewed.

When restoring a short clinical crown, the clinician may attempt to gain length by placing a subgingival margin. However, deep subgingival margins that encroach upon the biologic width jeopardize the periodontal tissue and are therefore not desirable (2,3). The short clinical crown cannot be evaluated by visual inspection alone. A thorough examination that includes clinical examination, radiographic examination, and diagnostic cast analysis is essential for successful rehabilitation of severely complicated oral dentition. Inadequate diagnosis and improper treatment plan may not ensure a satisfactory result. Visualization of the desired result is a prerequisite of successful therapy.

Crown retention and resistance form are primarily related to crown length, total occlusal convergence degree, and axial surface area. Secondary retention and resistance form may be derived from boxes, grooves, or pins placed in solid tooth structure. The relationship of axial wall height to prepared tooth width also greatly influences crown retention and resistance form. The axial wall height must be great enough to prevent the rotation of the casting around a point on the opposite margin. A crown on a short tooth preparation has a greater tendency toward displacement than a crown on a tooth of the same axial wall height with a smaller diameter. Sound tooth structure should provide the principal source of retention. Foundation restorations (buildups) should not be relied upon because it is difficult to know how well the foundation itself is retained. It is important to place the finish line on a sound tooth structure to ensure a favorable prognosis for the restoration (2)

## Search strategy

A PubMed search of English literature was conducted up to February 2011 using the terms: short clinical crown, surgical crown lengthening, forced eruption, diagnostic wax up, alveoloplasty and gingivectomy. Additionally, the bibliographies of 5 previous reviews, their cross references as well as articles published in Periodontol 2000, Compendium Continuing Education of General Dentistry, Journal of Periodontology, Quintessence International, Jouranl of American Dental Association, New York State Dental Journal, Journal of Prosthetic Dentistry and International Journal of Periodontics Restorative Dentistry were manually searched.

## Causes of short clinical crowns

Common causes of short clinical crowns (SCC) include (4-9).

Disease (caries, erosion, tooth malformation).

Trauma (fractured teeth, attrition).

Iatrogenic dentistry (excess tooth reduction, large endodontic access openings).

Eruption disharmony (insufficient passive eruption, mesially tipped teeth).

Exostosis, and genetic variation in tooth form.

## Restorability assesssment of short clinical crown

However, before any treatment is initiated on a tooth with short clinical crown, restorability must be established (4). Restorative assessment should include:

Consideration of the arch position of the tooth.

Strategic value of the tooth.

Periodontal considerations.

Crown-to-root ratio.

Interarch space occlusion.

Endodontic treatment feasibility.

Esthetics (2).

## Subgingical margin placement: A solution or problem to Short clinical crown

When restoring a tooth with a SCC, the clinician may attempt to gain length by placing a subgingival finish line. However, deep subgingival margins that encroach upon the biologic periodontal width (10-11) jeopardize the periodontium and are therefore not desirable. The biologic width-approximately 1 mm of epithelial attachment and 1 mm of connective tissue attachment is a physiologic entity that should not be violated. Invading the biologic width during tooth preparation can result in chronic inflammation, loss of alveolar bone, gingival recession and periodontal pocket formation. The amount of pathologic response is related to the individual patient’s susceptibility to periodontal disease (11-21). The chronic inflammation resulting from violation of the biologic width compromises both esthetics and periodontal health. Subgingival margins make adequate margin placement difficult, compromise provisional restoration maintenance, complicate impression making procedures and may preclude accurate evaluation of the final restoration and isolation to cementation (14).

## Other treatment options for short clinical crowns

The restoration of teeth with SCC has included the following techniques.

Alteration of tooth preparation design and placement of auxillary retention and resistance form features.

Placement of foundation restorations.

Surgical crown lengthening.

Orthodontic eruption.

Endodontic treatment and overlay removable partial dentures (2).

1. Altering tooth preparation design:

The short clinical crown does not permit the use of routine tooth preparation design. Additional design features are required to compensate for decreased retention and resistance form. Retention and resistance factors in tooth preparation are related primarily to surface area and height of the preparation, axial wall convergence, texture of the prepared surface and secondarily to intracoronal retentive devices (15-18). The walls of a short clinical crown should be as parallel as possible. Increased tapering of the walls decreases resistance and retention form, necessitating the use of secondary retention factors (pins, grooves, or boxes).

2. Placement of foundation restorations:

Increased crown length can be gained by the addition of foundation materials. Such foundation restorations may also be used to eliminate voids, undercuts, and irregularities in the tooth preparation. Foundation restorations can be classified according to both the tooth vitality and the means of foundation retention. The retention methods for vital teeth are chemical adhesion, micromechanical retention, pins, and grooves. For nonvital teeth the means of retention include both prefabricated and cast posts and cores, in addition to the methods used for vital teeth (2).

Teeth that have undergone endodontic therapy and have lost most or all coronal structure will require a post to retain a core for support and retention of the final cast restoration. Ideally, the optimal post length should be atleast equal to that of the clinical crown. A minimum 3 mm apical seal should be left after creating the space for the post. From this it can be determined that the effective root length supporting the clinical crown should be at least 3 mm longer than the clinical crown. Any length less than this will require a shorter post (a minimal apical seal of 3 mm must be left untouched) (19).

3. Surgical crown lengthening:

For the restorative dentist to utilize crown lengthening, it is important to understand the concept of biological width, indications, techniques and other principles.

Biological width: Biological width is defined as the dimension of space that the healthy gingival tissues occupy above the alveolar bone (20). Average biologic width equals epithelial attachment (0.97 mm) plus connective tissue attachment (1.07 mm). This gives an average value of 2 mm. Biologic width varies between teeth. Molars have greater biologic width than anterior teeth (21). The significance of biologic width to the restorative dentist has been well documented. Maynard and Wilson demonstrated a progressive inflammation with down-growth of the epithelial attachment and loss of connective tissue attachment as a result of violation to biologic width (11). When biological width is violated, as a defense mechanism, inflammatory response triggers alveolar bone resorption to provide space for a new connective tissue attachment, which results in increased pocket depth (22). A recent study by Lanning et al. was carried out on 18 teeth from 18 patients that required crown lengthening (23). Teeth were checked after surgery, at three and six months. The authors concluded that the preoperative measurement of biological width was reestablished after six months from the surgery, and, at the same time, an average of 3 mm of coronal tooth structure was gained as a result of the surgery. These results indicate that natural, healthy, periodontal biological width can be successfully obtained despite the respective nature of the procedure.

Attached gingiva: Several studies have shown that 2 mm to 3 mm band of attached gingiva is preferable to maintain the restored tooth successfully (24-25). It is of utmost importance when planning surgical crown lengt-hening to evaluate and measure the attached gingiva. Because of the resecting nature of this procedure, there is the risk of reducing the width of attached gingiva. Proper surgical technique-apically repositioning the full thickness flap-may even increase the band of attached gingiva, despite the resective surgery (20).

Based on these dimensions, Ingber et al. (12) stated that during clinical crown extension surgery

sufficient bone should be resected to permit 3 mm of sound tooth structure above the crest of bone: Rosenberg et al. (4) preferred 4 mm of tooth exposure. This bone resection is necessary to accommodate the supra crestal tissue, which will develop in the surgical site, and yet leave sufficient tooth exposed to complete the tooth pre-paration. In the absence of periodontal disease, transcrevicular probing, via the crevice to the crest of alveolar bone, may be used to determine dimension of the SGT at any specific site prior to surgery (26).

Indications:

Crown lengthening is indicated for teeth with reduced clinical crowns (20).

Clinical crown lengthening is indicated in these cases to gain additional tooth structures to meet the mechanical need of the restorative procedures (27).

Crown lengthening can also be indicated for biologic reasons, to prevent violation of biologic width and future attachment breakdown around the restored tooth.

In addition to providing sufficient tooth structure for functionally and biologically healthy restorations, crown-lengthening procedures are indicated for esthetic reasons. Among these reasons are when there are short teeth, excessive wear, uneven gingival contours, or a gummy smile (28).

Contraindications:

Teeth with deep carious lesions or fractures that result in non-restorable situations are contraindicated.

With restorable teeth, crown lengthening is contraindicated when there is an unfavorable crown-to-root ratio because of short roots or reduced bone support.

Exposure of furcation introduces potential periodontal breakdown and puts prognosis of the tooth in question (20). A recent study by Dibart et al. demonstrates that in molars, a preoperative distance of 4 mm between the furcation and the bone crest is needed to avoid the tooth at risk for furcation exposure (28).

Crown lengthening on a single anterior tooth causes uneven gingival contour, which is esthetically unpleasing, especially on patients with a high smile line (20).

Patients with debilitating systemic diseases or poor plaque control may compromise the healing potential and are contraindicated for surgical procedures (29).

Surgical considerations for Anterior teeth differ from those used posteriorly because of esthetic demands (30). The surgical result may be limited by anatomic factors (4,7) such as the location of the maxillary sinus, the vestibular depth, the position of the ramus and external oblique ridge, the amount of available keratinized tissue (especially on the distal aspects of the mandibular molars), and the thickness of the periosteum (if lacking sutures are to be placed), if surgical procedures are required, gingival width and thickness must be considered. Adequate gingival width for intracrevicular margin placement is approximately 5 mm (11,31), including 2 mm of free gingiva and 3 mm of attached gingiva.

Posterior crown lengthening: During surgery the amount of sound tooth structure must be measured circumferentially with a periodontal probe: there must be 5 mm of tooth structure coronal to the alveolar crest, 2 mm of tooth structure to maintain the biologic width, 1 mm of tooth structure to maintain the sulcular depth, and 2 mm of tooth structure for minimal retention and resistance form (2).

Anterior crown lengthening: A maximally esthetic anterior result can be achieved only if the set tissue contours blend from tooth to tooth (30,31-33), For placement of an intracrevicular restoration, there must be 5 mm of tooth structure incisal to the alveolar bone crest, including 2 mm to maintain the biologic width, 2 mm of sulcular depth for intracrevicular margin placement, and 2 mm for retention, with the finish line 1 mm into the sulcus. When the flap is sutured it should be placed 4 mm coronal to the alveolar bone crest. Tooth preparation should be performed 4 to 6 weeks after surgical crown lengthening for a supragingival finish line and 8 weeks after crown lengthening if the margins are to be placed in the sulcus (2).

4. Orthodontic Eruption:

There are many problems associated with clinical crown lengthening procedure. These are:

Following removal of bony support, an inverse and unfavorable root-to-crown ratio (R/C) can be expected due to the resultant long clinical crown.

If the osseous support of the tooth is questionable before surgery, additional removal of bone further decreases the R/C so that restoration becomes impractical.

Removal of supporting bone from adjacent teeth to create a normal bony architecture may severely compromise these teeth.

Should the ostectomy expose furcations, exceptional oral hygiene measures are needed to preserve the tooth.

Teeth that have short or conical roots may exhibit excessive mobility after surgery (19).

To overcome these drawbacks of clinical crown lengthening surgery, Heithersay (34) and Ingber (35) suggested the use of forced eruption for treatment of teeth with sound tooth structure at or below the bone crest, and for isolated osseous defects. The objectives include conservation of bone, preservation of biologic width, exposure of sound tooth structure for the placement of restorative margins, and maintenance of esthetics. For these reasons, the method of forced eruption is preferable.

Forced eruption or extrusion (36) is the intentional coronal displacement of a tooth, attachment apparatus (bone, connective tissue attachment, and epithelial attachment), and gingiva. Such therapy positions the root segment coronally, resulting in a more favorable crown-to- root ratio. In the absence of inflammation extrusion can progress 1 mm in 1 to 2 weeks. A lag period has been observed between the movement of a tooth and the movement of its attachment apparatus. The attachment apparatus and gingival unit follow the tooth after it begins to erupt from the alveolus. The faster the eruption is achieved, the longer the lag period. The degree of root tapering of single-rooted teeth is an important consideration, if the extruded root diameter is too small for the mesiodistal space between the adjacent teeth, an unesthetic embrasure space and overcontoured restoration will result (2).

Initiation of forced eruption: Before initiation of forced eruption, the restorability of the tooth after the orthodontic phase should be considered. The following steps are advised:

Estimate the length of the healthy root embedded in bone from the radiograph.

Estimate the space available for the clinical crown. Articulated diagnostic casts may be used as an aid.

Calculate the amount of eruption necessary to restore the tooth (4 mm sound tooth structure coronal to the alveolar crest).

Calculate the effective root length remaining after root extrusion and divide it by the clinical crown height as measured in step 2. If the result is 1 or more, then favorable conditions exist for completion of the restorative procedures. If the result is less than 1, then root extrusion will not provide the necessary basis for a properly constructed cast restoration (19).

5. Endodontic treatment and overlay removable partial dentures:

This is the economic and simplest treatment option. After endodontic treatment, the patient can be treated with removable overlay partial denture (2).

## Prosthodontic considerations while restoring a clinical crown

Under preparation of the tooth should be avoided, as an underprepared tooth inevitably results in an overcontoured crown.

The most apical extent of the full coverage restorations should not exceed the depth of the sulcus, even though it is not possible for the clinician to identify the most coronal extent of the junctional epithelium when prepa-ring a tooth (37).

The goal of establishing the finish line for a tooth preparation is based upon the retention of the retainer (38) and the provision of adequate space for the restorative cosmetic materials.

Chamfer preparations are necessary to provide the room for the cosmetic material of a restoration, there is usually no apparent reason for more than minimal extension of perhaps 0.5 to mm below the gingival crest.

When restoring elongated posterior teeth, the cosmetic material is not as critical, and the space for it can be provided in the design of the casting, rather than in the tooth preparation. Therefore, a full shoulder or deep chamfer tooth preparation is usually not necessary for elongated posterior teeth (37).

Marginal deformation has been repeatedly shown when a 1 mm collar is placed on a feathered edge preparation (39). This is not a factor for a molar full-gold cast crown or any posterior restoration with a 2-3 mm gold collar.

There is frequently a disparity between the apical extent of a restoration interproximally and radicularly. The parabolic architecture of the anterior teeth with their narrow alveolar process is more severe than the posterior teeth where the alveolar process widens to accommodate the larger root surfaces.

Inexperienced clinicians may mistakenly extend the tooth preparation on all surfaces to one circumferential depth, and this is likely to violate the interproximal soft tissue attachments of the periodontium. It is imperative not to commit this error, as it results in the extension of the interproximal margin too far subgingivally.

It is not important which impression technique is utilized. It is important to respect the fragility of the junctional epithelium and the attachment of the supracrestal fibers and to be careful not to disrupt them.

After the impression is secured and the die constructed, the next critical step is the demarcation of the finish line. This is referred to generally as “ditching the die,” and can be most precise only when accomplished by the same person who prepared the tooth. It is not possible to extend a casting too far apically if the die is properly ditched. This, then, precludes damage to the soft tissue attachment apparatus when trying on a casting or the framework for a fixed bridge.

When the restorative margin extends too far subgingivally, it may retain excess cement on its margin. This can be a plaque problem, and can result in an inflammatory response, as it may not be possible to remove the excess cement (37).

Considerations Post-Crown lengthening surgery for Supra Gingival Tissue (SGT) and preparation margins of restorations:

Previous studies indicate that the postsurgical dimension of SGT can vary. However, it is most likely that the amount of SGT formed will approximate the amount present prior to the surgery. Thus, once the preoperative amount of SGT present in a healthy operation site or in a contralateral area in the same individual is known then the situation of the preparation margin can be determined. During surgery when the flaps are replaced at or apical to the level of the alveolar crest, the gingiva will creep in a coronal direction until the full dimension of the predestined SGT is formed. Thus, if the dimension of the SGT for a given situation is known, it is possible to reliably predict the final position of the gingival margin that will be attained in approximately 1 year. If the final tooth preparation is contemplated within the first year after surgical crown extension, the preparation margin should not immediately be placed subgingivally. If it is placed immediately, as the SGT redevelops, the preparation margin can easily end up being located too far subgingivally. This is generally biomorphologically unacceptable, and the stage is set for progressive periodontal breakdown.

On the other hand, it is possible to utilize these events to advantage and carry out final tooth preparation, placing the preparation margins minimally coronal to the gingival margin 3 to 6 months postsurgically (26). The creeping of the SGT to its predestined dimension would ensure that the preparation margin ends up in an acceptable subgingival location. It is thus obvious that, in these situations in which preparation margins must be placed in the gingival crevice, it would be wise to delay the final preparation as long as possible. When this is not possible, the creeping attachment phenomenon may be used advantageously (40).

13. Esthetic crown lengthening requires careful treatment planning which includes determination of the desired gingival margin and bone level. Diagnostic wax-up and resin mock-up are useful tools. They provide an esthetic preview and also facilitate fabrication of a surgical template to record the desired gingival/bone location and guide the surgeon in identifying the exact location and amount of alveoloplasty/gingivectomy required. It is an invaluable tool of communication between the prosthodontist and periodontist if the case is referred (20).

## Conclusion

Extension of the preparations subgingivally to attain better retention form may have adverse reactions in the periodontium and may compromise esthetics. In these cases, surgical enhancement of the clinical crown is generally necessary to provide a dimension of clinical crown that permits acceptable tooth preparation and fabrication of a restoration compatible with the surrounding supracrestal gingival tissues. The vertical dimension, centric relation, and occlusal plane must be determined first, followed by a diagnostic wax up which is essential for fixed prosthesis. The complications with the short clinical crown demand a circumspect treatment plan and proper sequencing of therapy to ensure an optimal result for both the patient and the clinician. Proper treatment sequencing is critical when a patient requires multiple fixed restorations in conjunction with a removable partial and complete denture. An accurate diagnostic and interdisciplinary approach is necessary for obtaining improved, conservative and predictable results in esthetically compromised areas, such as the anterior mandibular dentition (1). In establishing a biologic basis for tooth lengthening, the following principles should be considered:

Whenever possible, the finish line of the restoration should be determined prior to surgery.

When the above is not possible, the finish line should be anticipated at surgery.

Sufficient alveolar bone should be removed to permit the development of an acceptable dimension of SGT between the actual or anticipated finish line of the preparation and the alveolar crest.

Transcrevicular probing circumferentially prior to surgery, in healthy areas in the operation site or in contralateral areas, should be the gauge for estimating the space needed for developing the SGT compatible with individual patient requirements.

The degree and configuration of osseous scalloping is determined by the surface topography of the tooth.

Gingival form is dictated both by the osseous configuration and the surface anatomy of the tooth.

Within limitations, gingival form may be modified by altering the topography of the tooth. This applies both to natural or implant-restored dentitions.

Restorative procedures must not disrupt the epithelial attachment and the SGT

The emergence profile of the restoration must not disrupt the crevicular wall of the SGT.

Preparation margins must not irreversibly disturb the dentogingival relationship, need be no more than 0.5 mm subgingivally, and should always parallel the gingival margins.

The final preparation and the restoration should be delayed as long as possible after tooth lengthening to permit the gingival margin to attain its predestined situation.

Where possible, preparation margins should be kept supragingival (26).
